# Academy of Stomatology

**Published:** 1899-04

**Authors:** 

**Affiliations:** Academy of Stomatology


### ACADEMY OF STOMATOLOGY.

A regular meeting of the Academy of Stomatology was held on Tuesday evening, December 27, 1898, at the rooms of the Academy, 1731 Chestnut Street, with the President, Dr. M. H. Cryer, in the chair.

A paper entitled "Clinical Studies of some Suppurative Diseases of the Maxillæ" was read by Professor H. C. Boenning.

(For Professor Boenning's paper, see page 146.)

DISCUSSION.

*Dr. M. H. Cryer.*—I was asked by the Academy to open the discussion upon this paper of Dr. Boenning's, and before doing so I wish to congratulate the Academy on having had such a paper read before it. As I understand it, the main point, not only in alveolar abscess, but in all abscesses, is to reach the part affected, and open it sufficiently. We can scarcely make the opening too large, and are thus able to remove not only the contents of the cavity, but its surroundings. In all abscesses, whether they be in the soft tissue or bone, it is not only the pus-cavity that is involved, but also the surrounding membrane, which used to be called the pyogenic membrane. In the soft parts we have this membrane; really, the line of demarcation between the healthy and the diseased tissue, or nature's attempt to expel the latter. It is well to go beyond this,—go down to healthy tissue. As Dr. Boenning has said, it is impossible to evacuate large pus-cavities through the apical foramina of teeth. Here is a skull showing a central incisor surrounded by a large opening in the anterior wall of the process extending through to and involving the hard palate. To evacuate this pus-cavity simply through the tooth is impossible, although we have dentists by the hundreds who are constantly endeavoring to do this. I believe, as Dr. Boenning has said, it is better to raise a flap and take away the necrosed bone, both labially and lingually. Dr. Truman will recall a case that I showed him a few weeks ago, of a large palatal abscess which extended entirely through the alveolar process, so-called, and could be felt buccally. I say "so-called," because we have no true line of demarcation between the true maxilla and the process, and in this case the abscess extended up into the bone proper. In the treatment of the case I raised a flap and with a large-sized bur drilled out the diseased tissue from the palatal surface. I packed the parts with the ordinary sterilized gauze incorporated with subnitrate of bismuth. In some cases this is not as irritating as iodoform gauze.

The doctor has spoken of disease of the antrum arising from a tooth, or from an abscess forming independently of a tooth, and passing up into the antrum. He speaks of opening into the antrum well and freely. I endorse this, but I have seen the anterior wall of the antrum cut away, the alveolar process thoroughly curetted, and the patient dismissed, but in a few months return with a discharge into the mouth. Therefore, we must look for more obscure causes than diseased teeth, or disease directly within the antrum. By clinical observations, and by dissection, I have traced abscesses within the antrum to the frontal sinus, and have shown on the screen photographs of specimens at the University which demonstrate, I believe, that infectious matter has passed into the antrum from abscessed teeth and out through the opening into the hiatus semilunaris, and from that through the infundibulum into the frontal sinus, and thence been communicated to the brain. I have seen cases of meningitis produced by diseased teeth. Then, again, I have specimens showing where the opening on one side of the frontal sinus has been closed, the bone becoming perforated, the infected matter has passed into the antrum, and teeth have been lost through disease originating in the frontal sinus and especially in the anterior ethmoidal cells. We should have to pass farther up and cut away the septum between the antrum and the nasal chamber. If the rhinologists of to-day find a suppurative condition in the nasal chamber and antrum, they advocate making an opening from the antrum into the mouth, and, instead of directing this infected matter through the nasal chamber into the pharynx, they drain it into the mouth, making it, if you will allow me to use the vulgar term, a "sewer." This is bad surgery. I think even rhinologists should be able to treat the nasal cavity, the ethmoidal cells, and the frontal sinus, and treat them in such a way that they will not be compelled to turn this effete matter into the mouth. If the rhinologist will not do it, the oral surgeon must do it; we must not have this infection in the mouth.

I hoped that Dr. Boenning would speak of another class of chronic abscess as generally found to be difficult to treat. I refer to external abscesses of the lower jaw. In a case of an abscess opening upon the body of the jaw at the facial notch, concerning which all the attendants at the clinic of Professor Garretson disagreed as to a diagnosis of tuberculosis, abscess from the submaxillary gland, or necrosed bone, the patient was brought here before the Academy. Dr. Jack examined the patient, and asked if a certain tooth was vital. I told him it responded to heat and cold, and I thought it was, but because of his question I drilled into the tooth and found the anterior pulp filaments vital, while the posterior one was putrescent. After placing the rubber dam over the tooth, carbolic acid was pumped into the canal, and after working this for some time I found that the carbolic acid had gone down through the tissue. Cotton saturated with carbolic acid was packed tightly into the canal, sealed in with gutta-percha, and the patient dismissed. The carbolic acid oozed out of the opening near the facial notch, and did not cease oozing until the next morning. The patient recovered, and the trouble has not returned. I have two patients now under my care who for some time have had abscesses in this region near the facial notch. Each is caused by a devitalized tooth. In many of these cases, after the tooth is extracted and the parts within the mouth become cured, we still have a constant discharge externally. Dr. Boenning has given us the best way of treating, and that is, to make a free incision and find out what is the cause. If there is dead bone, remove it; if there is infection of any kind, remove it. I believe that is the radical and proper method.

Another case is that of a colored person who came to the University of Pennsylvania Hospital with a large abscess. She had been sent there by a surgeon to have a tooth extracted, or to have the teeth examined. The surgeon had operated externally by making a large incision, cutting down to and scraping the bone. He had treated the case for months without any cure. She could scarcely open her jaws. By digital examination I found that there was an impacted lower third molar, and that the upper third molar was occluding upon the overlying gum tissue. By using a curved probe I found that there was a cavity in the lower third molar leading to a putrescent pulp. This, then, was the origin of the abscess and the cause of the trouble. The mouth could not be opened sufficiently to extract the third lower molar, but there was room enough to get a small universal root-forceps over the upper molar. It was removed, which, of course, relieved the gum tissue from occlusal irritation. In two weeks' time the jaw was opened sufficiently to pass a No. 3 elevator between the second and third molars, and by giving it a turn the latter tooth was removed. The socket immediately filled with pus, and by forcing peroxide of hydrogen into it disinfection was secured. I hope that by keeping this part clean the patient will get well without further operation.

*Dr. Truman.*—Notwithstanding that my good friend Dr. Cryer asserts that many dentists treat alveolar abscess through the canal, I believe that no intelligent dentist, unless wishing to give temporary relief, would now think of treating alveolar abscesses universally in that way. Now, the idea that alveolar abscess can be relieved or cured, not be recurrent, by any such treatment is, to my mind, almost an absurdity. What is an alveolar abscess? What are the symptoms? What are the phenomena following it? It stands exactly in the same relation to tissue as necrosed bone does to the maxilla. It is simply necrosed tissue. You cannot remove the pericementum from the tooth any more than you can the periosteum from the bone, and have remaining live tissue. Consequently, in all alveolar abscesses there is necrotic tissue always present as an irritant, and, therefore, there must be a constant source of recurrent trouble. As Dr. Boenning has said, the only real remedy for alveolar abscess is to cut off the necrosed root. It is a great mistake to go on treating a tooth by pumping carbolic acid or other agents into the canal, under the supposition that that will cure the diseased condition. I hold that it is never done, and do not see how it is possible. One might as well inject carbolic acid into a necrosed jaw and expect to have a return to health as to attempt to cure alveolar abscess by that kind of treatment. You must get at the sequestrum of the necrosed jaw, and you must remove the sequestrum of an abscessed tooth.

As to the antrum, Dr. Boenning unquestionably is right when he says it is better to make an opening as large as possible, if necessary, the better to wash out the antrum; but he takes it for granted, apparently, that the teeth have nothing to do with it. In my experience I have found that in almost every case the teeth have had something to do with it. The direction in which an alveolo-dental abscess will discharge will depend entirely upon whether or not the tooth-root has very nearly perforated the antrum, and we all know that very many of the roots do. Now, pus always follows the direction of least resistance, and if that is towards the antral cavity it will pass into it. Unless that tooth be removed, there will be a constant discharge into the antrum. Either wash it out or curette it, you still have the original cause, and I hold that in the treatment of all inflammations the cause must be found and removed; any other treatment is almost malpractice. There may be other causes of antral empyema, such as tumors and cysts, but if the cause is a dental one, and that is easily determined, the first essential is the removal of the tooth.

*The President.*—As Dr. Boenning is anxious to leave early, perhaps he would like to answer Dr. Truman.

*Dr. Boenning.*—I desire to call attention to a paragraph or two which seem to have been overlooked by the gentlemen in speaking of treatment. I said, following the thought laid down in the discussion of treatment of alveolar dental abscess, that "it occurred to me several years ago that if we desire to cure antral abscess, so called, we must establish an opening sufficiently large to thoroughly evacuate the antral cavity, clear out the purulent material, curette the carious walls of the antrum, and to completely remove all pathological masses or substances." If there is a diseased root in the floor of the antrum, what is that but a pathological condition? Therefore, instead of discussing at random the theme of the subject here, it is plain that a pathological condition is any diseased condition, whether it affects a tooth, a bone, or a soft structure, and if that presents in the floor of the antrum, then it is amenable to the same line of treatment as the removal of a necrotic root in the case of alveolo-dental abscess. I do not desire any one to imagine for one moment that in the treatment of cases of antral abscess I would have you open up the antrum, discharge its contents, remove the pus, and allow a necrotic root or roots, as the case may be, to remain. The speakers have merely misinterpreted the paper, which distinctly advocates the removal of the cause.

*Dr. Truman.*—I understood Dr. Boenning to say that he thought it was almost malpractice to remove a firm tooth.

*Dr. Boenning.*—A firm tooth, yes; by which I desire it to be understood a tooth which shall be to all intents and purposes a normally healthy tooth, free from pathological conditions. I will leave it to the discussion of the society as to whether a necrotic root is ordinarily firm or not.

*Dr. Cryer.*—I would like to show a specimen from a white subject, with the roots, both buccal and lingual, of a molar almost penetrating the antrum. This, I believe, is the case in nearly all of the white race, but not in the negro or the Japanese, as far as I have been able to ascertain. We find the roots of the teeth in this negro skull are far from the antrum. I believe that in the colored race we will seldom have the antrum affected by abscessed teeth, while in the white race we are much more liable to have it.

(Replying to question of Dr. Hickman.) I have not only seen the second, but first bicuspid root enter the antrum, and have seen a cuspid root in a position where, if an abscess were to occur, the infected matter could enter the antrum. A specimen at the University of Pennsylvania presents a condition where the apices of the roots of the teeth from the central and to the second molar, inclusive, are in the nasal chamber; in other words, the antrum does not come down into relation with the teeth, but is cut off above them, while the external wall of the nasal chamber passes over to the buccal side of the maxillary bone.

*Dr. Jack.*—Mr. President, in my treatment of primary alveolar abscess, I must say that it has always been conducted through the canals, and in a large majority of instances the treatment of such has been successful after the complete disinfection of the canals and thorough flushing of the roots, where this was possible.

With chronic abscesses my success has not been so pronounced, though in some instances, where I could get the passage of disinfectants through the canal and fistula, the treatment has been successful, unless there has been infection of a necrosed root. In one case, of a lower molar, there was an abscess continually discharging from the anterior root, though the canal had been correctly filled. The tooth at length became so extremely mobile that I felt convinced that the patient would lose the tooth, and so announced it to her. She replied, "I will not have the tooth removed, and you must cure it." I administered ether, and amputated the end of the root which was necrosed. In a few weeks the part became entirely healed, and there has been no recurrence.

In my experience of forty years I have had but two or three actual cases of antral empyema,—one about two years ago, and the other within a few weeks. The one two years ago occurred in the mouth of the wife of a physician. I had made application to destroy the pulp of a tooth, and had partially devitalized it, when the patient, on account of some physical condition, was sent by her husband to the sea-shore. While there the tooth became more seriously involved, the tissues became inflamed, and when she returned, very shortly afterwards, there was evidence of empyema of the antrum. The tooth was extracted immediately. Her husband, being a surgeon, took charge of the case. He opened the antrum immediately posterior to the canine fossa, and after treatment of some weeks he entirely cured the case.

Another case occurred in a lady patient about four years ago. A consultation was asked for by the husband, also a physician. After careful examination of the teeth by the thermal test, I found them all to contain vital pulps, and I gave it as my opinion that the teeth had to be excluded as the cause of the disturbance. The case then passed into the hands of the rhinologist, who took charge of the case, as an extension of a purulent nasal condition. The patient recovered. In that case there was a large amount of discharge and very great fœtor, but there was complete recovery.

*Dr. Jeffries.*—I was a long time ascertaining the origin of the worst antral abscess I have had to treat. There was in the mouth a devitalized molar; on the same side one of the bicuspids was loosened, and the other was devitalized. After drilling into the molar, which I supposed naturally was the cause, and getting an opening through the lingual root into the antrum, I treated through it for some time. Afterwards I opened through the antral wall, and, though the condition was ameliorated, it did not result in a cure. I opened the bicuspid and found that was not the cause, and, as a last resort, I took out an old filling, which had been for twenty years or more in the canine tooth, and found that it was responsible for the abscess; yet there was not a particle of external evidence that that tooth was involved. There was no soreness manifested; but after that was treated, together with the opening in the frontal aspect of the antrum, a cure was quickly effected. In another case an abscess over the roots of the central and lateral incisors reached back to the centre of the hard palate, where fluctuation could be distinguished. The treatment consisted of burring the necrosed bone and the apices of two teeth. The cavity, after several months of continuous treatment, filled up with granulations completely. The origin of the disturbance was in the central incisor, it having been devitalized and filled for probably twenty-five years. The treatment was tedious and recovery slow, on account of the physical condition of the patient, who was in poor health, but there has been no recurrence after cure was considered perfect.

*Dr. Hickman.*—I went with a physician to see an antral case concerning which he wished to consult me. I asked him if he had extracted any of the teeth, and he said, "Yes, all the front row." I asked him why he did not begin with the other end. He said, "Those teeth have no connection with the antrum whatever. I would not extract those teeth for anything in the world." The patient had three upper molars on each side. I gave my opinion, which was not acceptable, and some time later I heard of the patient's death.

*Dr. Darby.*—I think any one would infer from the paper this evening that all cases of chronic alveolar abscess required perforation of the alveolar plate. If that impression has obtained, I am certainly not one to endorse it. I know that a great many chronic alveolar abscesses are cured without perforation of the alveolar plate, and should be sorry to think that dentists were in the habit of performing the operation to cure ordinary chronic alveolar abscesses. Perhaps in many instances we might cure them as promptly, or more so, by cutting away the opening and evacuating what might be in the abscess cavity, but the ordinary cases we meet with, unless there may be necrosis, are amenable to escharotic and disinfecting medicaments. I saw, only a few years ago, a lateral incisor that had been suppurating for twenty years. The man said that every morning he pressed pus from a little sac on the mucous surface, and that was the end of it for that day. I opened the tooth and pumped wood creosote through the fistula. A cure was effected in twenty-four hours. I have seen the gentleman once a year since, and there has been no return, in spite of his sixty years and the fact that it was a chronic abscess of twenty years' standing. In the lower jaw we frequently meet with cases more difficult because the roots are more tortuous or more constricted. If we could pump medicaments through inferior molars, we should have less difficulty in curing. There are cases where I think we would do well to run a bur right through the alveolar plate and curette the diseased area, but in many cases there is no occasion for it. By filling these canals as thoroughly as I can, carrying a seton of iodoform gauze to the bottom of the fistula and letting it stay until it works out, I get healthy granulation from the bottom. Where there has been necrosis of the root apex, the only thing indicated is amputation. I endorse the free opening of the antrum when opening is indicated.

*Dr. James Truman.*—I think we ought to define our words somewhat. Dr. Darby uses the word "cure." I do not quite understand how he can use that word in connection with alveolar abscess. I recognize the fact that by pumping in creosote or carbolic acid, or any of the escharotics used, we can produce a toleration with a necrosed portion, but I cannot comprehend how we can have alveolar abscess in any tooth and the pericementum retain vitality and continue to perform its function. The very fact that we have pus in a given cavity necessitates a separation of the pericementum and periosteum of the bone, and we have what is ordinarily called a sac. Does that periosteum or pericementum reunite with the tooth at that portion? Not if I understand the philosophy of repair in teeth, or anywhere else in the organism. It remains always as a foreign body.

*Dr. Darby.*—If it lasts twenty years and gives no more trouble, it is then practically cured?

*Dr. James Truman.*—I cannot call that cured, but the tooth is simply placed in a state of tolerance.

*Dr. Register.*—I had a case of external abscess at the facial notch which was as large as a hen's egg. The patient had been treated for some time by a surgeon, who had failed to establish a dental origin. I found a devitalized molar, cleansed its canals, and pumped thirty per cent. of sulphuric acid through them into the abscess tract. I treated externally with dilute iodine and chloroform. It was healing rapidly, when the patient called upon and convinced his physician that the trouble was due to a tooth. His physician acknowledged his error, and told him to have the tooth out, which advice was acted upon without first consulting me. As I had given the patient his choice of treatment or extraction in the first instance, my annoyance at the professional discourtesy was very great. I prefer the dilute sulphuric acid in these cases, because of its great solvent power over necrosed bone. One day, while performing an implantation of a cuspid tooth, to my surprise I entered the antrum. I merely changed the direction of my bur, implanted the tooth, and all went well. I have operated upon a cat for extensive maxillary necrosis, packing the wound with iodoform gauze, stitching the cheek, and paying no further attention. It recovered.

*Dr. McCullough.*—I was asked several years ago by a veterinary surgeon to assist him in an operation on a dog. There was a fistulous opening on the line of the lower jaw. As he was doubtful as to the cause of the disease, I, with a probe, followed the canal in the direction of the first molar tooth. I was not satisfied that there was a perceptible discoloration of the tooth, but, such a cause not being uncommon in our practice, I recommended the extraction of the lower first molar, which resulted in a permanent cure.

Another case was that of a man, thirty-two years of age, who, when a boy of fifteen years, had the edge of an upper lateral incisor broken by the sudden letting go of a brake on a horse-car. Several years later he began suffering from neuralgic pains throughout the side of the face and head. He was treated continually internally by physicians, and was sent to me by his last adviser when he was about thirty-two. I found the lateral incisor very much discolored, a swelling of the gum in front of the apex of the root, and a pulsating sac of considerable size on the same side of the palate. I opened the tooth to prove my diagnosis, and, as the sac in the roof of the mouth was below the level of the apex of the root, I believed it impossible to force the contents of this sac up to and through the root by the apex. I therefore made an incision of about an inch over the swelling in the palate, when my lancet sunk in an opening of one-eighth of an inch in the palate bone. I then, with the syringe, forced different germicidal fluids through the root and out through the opening in the palate, and the reverse. I cleaned the tooth thoroughly and filled the root immediately with gutta-percha. I left a drain in the opening in the palate for several days. There is every evidence of an absolute cure. Two years have elapsed since the operation with no change.

Otto E. Inglis,
*Editor Academy of Stomatology.*

